# Pathogenic microbes, the microbiome, and Alzheimer’s disease (AD)

**DOI:** 10.3389/fnagi.2014.00127

**Published:** 2014-06-16

**Authors:** James M. Hill, Christian Clement, Aileen I. Pogue, Surjyadipta Bhattacharjee, Yuhai Zhao, Walter J. Lukiw

**Affiliations:** ^1^Department of Microbiology, Immunology & Parasitology, Louisiana State University Health Sciences CenterNew Orleans, USA; ^2^LSU Neuroscience Center, Louisiana State University Health Sciences CenterNew Orleans, USA; ^3^Department of Ophthalmology, Louisiana State University Health Sciences CenterNew Orleans, USA; ^4^Alchem BiotekToronto, ON, Canada; ^5^Department of Neurology, Louisiana State University Health Sciences CenterNew Orleans, USA

**Keywords:** Alzheimer’s disease, microbiome, virus replication, prion, miRNA

Alzheimer’s disease (AD) is a progressive neurodegenerative disorder and the leading cause of cognitive and behavioral impairment in industrialized societies. The cause of AD is unknown and the major risk factor for AD is age. About 5% of all AD cases have a genetic or familial cause however the vast majority of all AD cases (~95%) are of sporadic origin. Both the familial and the sporadic forms of AD share a common disease phenotype involving at least eight characteristic features including (i) evidence of uncontrolled oxidative stress; (ii) up-regulated pro-inflammatory signaling; (iii) changes in innate-immune signaling; (iv) the progressive accumulation of lesions including neurofibrillary tangles (NFT) and amyloid beta (Aβ)-containing senile plaques (SP); (v) significant synaptic signaling deficits; (vi) neurite and brain cell atrophy; (vii) progressively altered gene expression patterns that are different from healthy brain aging; and (viii) progressive cognitive impairment and dementia in the host. There is currently no cure or adequate clinical treatment for AD, and it remains unclear how AD originates and propagates throughout the brain and central nervous system (CNS). Results from recent genome-wide association studies (GWAS) indicate that a significant portion of AD-relevant gene signals are not located within gene coding regions suggesting the contribution of epigenetic or environmental factors to AD risk. The potential contribution of pathogenic microbes to aging and AD is becoming increasingly recognized (Miklossy, 2011; Cho and Blaser, 2012; Bhattacharjee and Lukiw, 2013; Poole et al., 2013; Heintz and Mair, 2014; Huang et al., 2014; Mancuso et al., 2014). Importantly, most of the changes seen in AD, such as inflammation, brain cell atrophy, immunological aberrations, amyloidogenesis, altered gene expression and cognitive deficits are also seen as a consequence of microbial infection (Cho and Blaser, 2012; Yatsunenko et al., 2012; Bhattacharjee and Lukiw, 2013; Foster and McVey Neufeld, 2013; Kim et al., 2013; Heintz and Mair, 2014; Mancuso et al., 2014). This brief communication will review some recent observations on the potential contribution of pathogens to neurological dysfunction, with specific reference to AD wherever possible.

Firstly, humans contain a complex and dynamic community of microbes called the microbiome that forms a “metaorganism” with symbiotic or commensal benefit to the host (Cho and Blaser, 2012; Bhattacharjee and Lukiw, 2013; Heintz and Mair, 2014). The microbiome of the human gastrointestinal (GI) tract contains the largest reservoir of microbes, containing about 10^14^ microbes from at least 1000 distinct microbial species, and outnumbering human host cells by about 100 to 1 (Whitman et al., 1998; Kim et al., 2013). The GI tract microbiome has been estimated to encode about 4 × 10^6^ genes so the quantity of the microbiome genes outnumbers host genes by about 150 to 1 (Bhattacharjee and Lukiw, 2013). Over 99% of GI tract microbiota are anaerobic bacteria, with fungi, protozoa, archaebacteria and other microorganisms making up the remainder; interestingly only two bacterial divisions are prominent in GI tract microbiota, including *Firmicutes* (~51%) and *Bacteroidetes* (~48%), with the remaining 1% of phylotypes distributed amongst the *Cyanobacteria, Fusobacteria, Proteobacteria, Spirochaetes*, and *Verrucomicrobia*, with various species of fungi, protozoa, viruses and other microorganisms making up the remainder (http://www.genome.gov/pages/research/sequencing/seqproposals/hgmiseq.pdf). Of all human GI tract microbiota, bacterial densities of up to 10^12^ per ml are the highest recorded density in any known microbial ecosystem (Whitman et al., 1998; Bhattacharjee and Lukiw, 2013; Kim et al., 2013). Interestingly, the microorganisms making up the smallest 1% of the microbiome have a disproportionately large effect on disease, and it is a major function of the healthy GI tract microbiome to keep under control the proliferation of any potentially pathogenic microbes contained within (Hornig, 2013; Kim et al., 2013; Heintz and Mair, 2014; see below).

Recent interest in the role of the microbiome in human health and disease has rapidly expanded over the last several years with the advent of new sequencing and bioinformatics technologies for interrogating the genetics of complex microbial communities and microbial-host interactions. There is currently much interest in the ability of GI tract bacteria to influence host innate-immune, neuroinflammatory-, neuromodulatory- and neurotransmission-functions (Bravo et al., 2012; Bhattacharjee and Lukiw, 2013; Boutajangout and Wisniewski, 2013; Brenner, 2013; Douglas-Escobar et al., 2013; Heintz and Mair, 2014; Mancuso et al., 2014). While potentially pathogenic GI tract microbes are kept in check by a homeostatic commensalism, their increased abundance has been associated with diseases that include anxiety, autoimmune-disease, diabetes, metabolic-syndrome, obesity, and stress-induced and progressive neuropsychiatric diseases including autism, schizophrenia and AD (Bravo et al., 2012; Bhattacharjee and Lukiw, 2013; Boutajangout and Wisniewski, 2013; Foster and McVey Neufeld, 2013; Hornig, 2013; Heintz and Mair, 2014).

Here we list 10 recent, highly specific and illustrative insights into the potential contribution of pathogenic microbes, altered microbiome signaling and other disease-inducing agents to the development of AD:

1. **Fungal infection of the CNS:** AD is characterized in part by amyloid-containing senile plaques (SPs) and neurofibrillary tangles (NFTs) that induce microglial cell activation, neuroinflammation and brain and neurovascular cell dysfunction. Recently yeast and fungal proteins including (1,3)-β-glucan, high levels of fungal polysaccharides and disseminated and diffuse mycoses in the peripheral blood of AD patients suggests that chronic fungal infections may increase AD risk (Prusiner, 2013; Alonso et al., 2014; Heintz and Mair, 2014). It is interesting that amyloids are associated with the surface structures of fungi which may aid in their organization and promote higher order assembly of amyloid (Liu et al., 2008; Asti and Gioglio, 2014).
2. **HSV-1 is associated with AD:** Abundant evidence suggests that the double-stranded DNA (dsDNA) herpes simplex virus-1 (HSV-1), a neurotrophic, neuroinvasive member of the family Herpesviridae can establish lifelong latency in CNS tissues and contribute to AD (Jamieson et al., 1991; Kammerman et al., 2006; Itzhaki and Wozniak, 2008; Toma et al., 2008; Lukiw et al., 2010; Ball et al., 2012; Agostini et al., 2014; Mancuso et al., 2014). A particularly interesting observation is that when human brain cells are infected with HSV-1, there is a significant and selective up-regulation in the expression of a small non-coding single-stranded RNA (ssRNA) known as miRNA-146a. This pro-inflammatory miRNA is also significantly up-regulated in anatomical areas of the human brain affected with AD and in human prion diseases where it contributes to altered innate-immune responses (Lukiw et al., 2010; Prusiner, 2013). It is noteworthy that of all ~2000 known miRNAs the same inducible, pro-inflammatory miRNA-146a is specifically up-regulated in both AD and in HSV-1 infected brain cells (Hill and Lukiw, 2014). Besides host-encoded miRNAs, ~200 virally encoded miRNAs have been discovered in dsDNA viruses (mainly HSV-1 and polyomoviruses)—these regulate fundamental biological processes including immune recognition, promotion of cell survival, angiogenesis, cell proliferation and differentiation, but their contribution to the AD process is not well understood (Plaisance-Bonstaff and Renne, 2011).
3. **Prion diseases:** driven by an unusual type of self-replicating “microbe,” prion diseases are sporadic, inherited or acquired and ultimately fatal neurological disorders highly similar to AD (Lukiw et al., 2011; Prusiner, 2013). Aβ peptide “prion-like” aggregates induce widespread amyloidogenesis after inoculation into susceptible animal hosts (Stöhr et al., 2012; Prusiner, 2013). The recent discovery that prions can serve as Aβ receptors to relay amyloid neurotoxicity, and that peripherally administrated prions reach the brain, has engendered renewed interest in this self-replicating protein and its involvement in AD-like signaling processes that include neuroinflammation, synaptic degeneration and amyloidogenesis (Prusiner, 2013; Chen et al., 2014; Hernandez-Rapp et al., 2014).
4. ***Chlamydophila pneumoniae*, other pathogenic bacteria and AD**: The association of the gram negative, obligate intracellular bacteria and pneumonia-causing *C. pneumoniae* of the family *Chlamydiaceae* with diseases such as coronary artery disease, arthritis, multiple sclerosis, meningoencephalitis, and AD has recently gained serious attention (Balin and Hudson, 2014; Wunderink and Waterer, 2014). Atypical extracellular *C. pneumoniae* antigens in the neocortex of AD brain and their association with SP and NFT suggest the contribution of *C. pneumoniae* infection to AD pathology (Hammond et al., 2010; Choroszy-Król et al., 2014). It is interesting to note that virtually all AD patients expire from pneumonia as a cause of death and not as a direct consequence of AD itself (Choroszy-Król et al., 2014; Huston et al., 2014). Interestingly, other recent studies have further implicated *Borrelia* species, *Helicobacter pylori*, the periodontopathic spirochaete *Treponema denticola, Tannerella forsythia, Porphyromonas gingivalis* and other bacteria with increased incidence of age-related dementias including AD (Miklossy, 2011; Poole et al., 2013; Huang et al., 2014).
5. **HIV-1 and AD:** Human immunodeficiency virus (HIV), a lentivirus of the family *Retroviridae*, is a slowly replicating virion containing a 9749 nucleotide ssRNA genome that causes acquired immunodeficiency syndrome (AIDS)—a condition in which progressive failure of the immune system allows opportunistic infection (Borjabad and Volsky, 2012; Risacher and Saykin, 2013). HIV-associated neurocognitive disorders (HAND) is a common manifestation of HIV infection and encompasses a variety of neurological disorders including AIDS dementia complex, HIV-associated encephalopathy and AIDS-associated cognitive decline (Borjabad and Volsky, 2012; Widera et al., 2014). Histopathologically HIV-infected brains exhibit atrophy of neurites and neuronal loss in anatomical areas identical to what is seen in AD (Widera et al., 2014). Recent comparative meta-analysis further indicate that brains of patients with HAND and AD share common mis-regulated gene expression profiles implicating altered neuroimmune responses and progressive deficits in synaptic transmission (Borjabad and Volsky, 2012). Conversely, Aβ42 peptides appear to enhance HIV-1 attachment and entry to promote productive infection in susceptible cells of the CNS (Widera et al., 2014).
6. ***Toxoplasma* and neurodegeneration**: *Toxoplasma* species such as *Toxoplasma gondii* are intracellular protozoan parasites that can cause encephalitis and neurological dysfunction by promoting chronic inflammation of the brain and CNS. Recently AD has been associated with significantly increased anti-*T. gondii* antibodies suggesting a possible mechanistic link between *T. gondii* infection and AD (Prandota, 2014).
7. **Viroids, miRNAs and AD:** viroids are minimalist plant pathogens that consist of a viroid-specific ssRNA that are remarkably similar to miRNAs in their mode of generation, processing, structure and function, mobility and ability to spread disease within the host (Hill et al., 2009; Hill and Lukiw, 2014; Pogue et al., 2014). Abundant research evidence now strongly links miRNA dysregulation with AD and represents a new class of biomarkers which may be diagnostic for AD (Alexandrov et al., 2012; Danborg et al., 2014; Maffioletti et al., 2014). We may be able to gain insight on the mechanism of AD neuropathology driven by miRNA from what is already known about plant viroids and their ability to spread systemic degenerative disease (Navarro et al., 2012; Hill et al., 2014; Pogue et al., 2014).
8. **Hepatitis and AD:** Hepatitis C virus (HCV) is a small, enveloped ssRNA virus of the family *Flaviviridae* containing a ~9600 nucleotide genome that causes a chronic infectious disease of the liver and is associated with neuroimmune disorders (Monaco et al., 2012). HCV infection has recently been shown to significantly increase the risk for AD, especially in the aged (Chiu et al., 2013; Karim et al., 2014).
9. **Cytomegalovirus and AD**: A growing number of common viruses and latent viral infections involving *Herpesviridae* have been linked to the development of AD, and one of these is the human cytomegalovirus (HCMV). HCMV infection is often unnoticed in healthy people, but can be life-threatening for immune-compromised, HIV-infected or organ transplant patients. Recently blood serum, cerebrospinal fluid, and cryopreserved lymphocytes from AD patients were analyzed for associations between CMV infection and AD and it was found **(i)** that CMV antibody levels were positively associated with the number of NFT; **(ii)** that the percentage of senescent T cells was significantly higher for CMV-seropositive as compared to CMV-seronegative subjects, and **(iii)** that this was associated with the pathologic diagnosis of AD (Lurain et al., 2013).
10. **GI tract and blood-brain barrier permeability**: Lastly and importantly, the GI tract epithelial barrier and the blood brain barrier both become significantly more permeable over the course of aging (Brenner, 2013; Tran and Greenwood-Van Meerveld, 2013). This may make the CNS more susceptible to potential neurotoxins generated by microbiome-resident or environmental pathogens. Environmental and dietary influences, including chronic bacterial or viral infections can progressively alter blood-brain barrier permeability and thereby facilitate cerebral colonization by opportunistic pathogens as we age (Welling et al., 2014).

Taken together, it is clear that the human CNS is under constant assault by a wide array of extrinsic and intrinsic neurotrophic microbes and pathogens including bacteria, virus, fungus, nucleic-acid free prions, or small non-coding RNAs found both in the environment and contained within the microbiome. *Virtually every type of microbe known has been implicated in contributing to the susceptibility and pathogenesis of the AD process*. This may be especially important over the course of aging because innate-immune and physiological barriers are often compromised with age, enabling microbes and/or their ‘neurotoxic secretions’ to gain easier access to CNS compartments (Brenner, 2013; Tran and Greenwood-Van Meerveld, 2013; Welling et al., 2014). Because AD is clearly a multifactorial disease, and there are multiple biological pathways by which brain cells can dysfunction, perhaps it is not too surprising that multiple and complex microbial insults could contribute to AD, including the spreading of pathological signals throughout the CNS (Alexandrov et al., 2012; Bhattacharjee and Lukiw, 2013). *The contributions of microbes to multiple aspects of human physiology and neurobiology in health and disease have up until now not been fully appreciated.* Interestingly, both the entorhinal cortex-hippocampal axis and the olfactory system have been suggested as the earliest anatomical regions targeted by AD, indicating that perhaps primary as well as opportunistic infections might contribute to AD pathogenesis (Lazarov and Marr, 2010; Bhattacharjee and Lukiw, 2013; Cross et al., 2013; Balin and Hudson, 2014). What is currently known is (i) that most neurological disorders have a progressive, age-related and geographical character; (ii) that the composition of the human microbiome and exposure to pathogens changes with age, diet, lifestyle, and biological environment; and (iii) that microbial exposure, microbiome complexity and AD incidence are highly variable in different human populations (Yatsunenko et al., 2012; Lukiw, 2013; Danborg et al., 2014; Heintz and Mair, 2014). Intelligent pharmacological approaches including anti-bacterial, anti-viral and/or anti-inflammatory drugs in combination, or drug strategies more effectively directed toward the health and homeostasis of the holobiome should be useful in the future clinical management of AD and related degenerative disorders with an immune and inflammatory component.

## Conflict of interest statement

The authors declare that the research was conducted in the absence of any commercial or financial relationships that could be construed as a potential conflict of interest.
